# Barriers and facilitators to diagnosing and managing apathy in Parkinson’s disease: a qualitative study

**DOI:** 10.1186/s12883-019-1329-z

**Published:** 2019-05-24

**Authors:** Bria Mele, Zahra Goodarzi, Heather M. Hanson, Jayna Holroyd-Leduc

**Affiliations:** 1 Department of Community Health Sciences, University of Calgary, Foothills Medical Centre, South Tower, Room 1104, 1403-29 St. NW, Calgary, AB T2N 2T9 Canada; 2 Department of Medicine, University of Calgary, Calgary, Alberta Canada; 3 Alberta Health Services, Calgary, Alberta Canada; 4 Seniors Health Strategic Clinical Network, Alberta Health Services, Calgary, Alberta Canada; 5 Departments of Medicine and Community Health Sciences, University of Calgary, Hotchkiss Brain Institute, O’Brien Institute for Public Health, University of Calgary, Calgary, Alberta Canada

**Keywords:** Parkinson’s disease, Apathy, Diagnosis, Management, Qualitative research, Framework analysis

## Abstract

**Background:**

Apathy is a prominent non-motor symptom in Parkinson’s disease (PD). People with apathy show a lack of emotion, passion, and motivation. Between 17 and 70% of persons with PD have apathy; the extreme heterogeneity in these estimates is due to limited heterogeneous knowledge concerning how to diagnose PD. The lack of a widely utilized diagnostic process limits understandings on how to treat and manage apathy in PD. A scoping review of apathy in PD identified only one qualitative study investigating this symptom. It was our objective to assess perceived barriers and facilitators to diagnosing, treating, and managing apathy in PD, as described by key stakeholders.

**Methods:**

This research applied qualitative methodology, utilizing focus groups and interviews with health care practitioners (HCPs), persons with PD, and caregivers. Evidence gathered from a scoping review on apathy in PD informed discussions that took place with participants. Data collection and analysis was conducted using framework analysis, applying the Theoretical Domains Framework and Behaviour Change Wheel.

**Results:**

Eleven HCPs and five persons with PD/caregivers participated. Themes included interdisciplinary teams and communication with family to facilitate diagnosis and treatment, and the use of education and increased awareness of apathy to facilitate management. Themes surrounding barriers included lack of initiative and motivation to maintain treatment plans, and a lack of evidence for apathy specific interventions. While a key barrier identified was the lack of information HCPs have access to, persons with PD and caregivers would prefer to receive a diagnosis of apathy even with limited management methods. Thus, education and awareness were noted as two of the most important facilitators, overall.

**Conclusion:**

These findings suggest that diagnosing, treating, and managing apathy in PD requires interdisciplinary teams, that include family and caregivers. We identified that where HCPs perceive lack of knowledge as a barrier to diagnosis, persons with PD and caregivers find being given a diagnosis facilitates understanding. These findings highlight the importance of qualitative research involving persons with PD and apathy, caregivers, and HCPs who aid in management of this symptom. Barriers reported suggest future research must aim to identify apathy specific treatments, both pharmacologic and non-pharmacologic.

## Background

Apathy is present in 17–70% of persons with Parkinson’s disease (PD) [1, 2]. It is primarily conceptualized as a dysfunction in motivated behavior, causing individuals to feel a lack of emotion, passion, and motivation [3, 4]. Apathy has also been conceptualized as a disorder of self-initiative and motivation affecting emotion, cognition, and overt behaviour [4]. Apathy contributes significantly to poor quality of life in those with PD, compared to other non-motor symptoms [5, 6].

Despite how common apathy is, there are no universally accepted diagnostic criteria, however several diagnostic criteria have been proposed [7, 8]. While such diagnostic criteria have been validated within PD populations, recent updates have been published and require further validation [7, 9]. Furthermore, the lack of universally accepted diagnostic criteria results in variation in how screening tools to detect apathy in PD are validated. The variation in assessment of psychometric properties results in heterogeneous understandings of apathy within the literature [2]. This subsequently limits the information and support given to persons with PD and their family caregivers [10]. As family caregivers often provide care for individuals with PD, this symptom affects more than those with PD and is an important focus for research [11, 12].

The lack of widely utilized diagnostic criteria for apathy in PD is a key problem surrounding the diagnosis and treatment of this symptom [13, 14]. PD specific apathy rating scales have been developed and validated within PD populations [13–15]. However, these scales have not been validated using an accepted definition for apathy [14, 16]. Given the heterogeneity associated with the validation of available apathy screening tools, they have not been consistently adopted across the literature [2, 15]. Research highlights the importance of validating such tools against an accepted gold standard criteria for apathy [15]. Thus, the current lack of consensus regarding a definition and approach to diagnosis remains an important gap within the literature.

Treatment options for apathy in PD are also lacking [17, 18]. In a preceding scoping review, our team synthesized all available literature on diagnosing and managing apathy in PD [19]. This synthesis provided an understanding of what is currently known, and where gaps exist. Of the 323 included studies on apathy and PD, there were no conclusive findings regarding effective treatment for apathy in PD, either pharmacological or non-pharmacological [20].

As gaps within the literature have been clearly identified, research must assess why these gaps exist and determine the facilitators and barriers to care. This will ensure meaningful future steps are taken to improve the quality of care provided to those with PD and apathy. Therefore, our primary objective was to assess patient, family caregiver, and health care practitioners (HCP) perceptions around the barriers and facilitators to diagnosing and managing apathy in PD.

## Methods

### Design and ethics

This study employed semi-structured focus groups and interviews and used framework analysis to analyze data. Both focus groups and interviews were employed to ensure flexibility with participant schedules. Ethics approval was granted through the Conjoint Health Research Ethics Board (CHREB-17-0669). All researchers involved and participants signed confidentiality agreements and informed consent.

### Context, setting, and sampling

The use of theoretical thematic analysis, applying the Theoretical Domains Framework (TDF) and Behaviour Change Wheel (BCW), ensured evidence and interventions were directly linked. We estimated between four to six individuals per stakeholder group would be required to reach saturation [21].

Participants were divided into two groups: i) HCP including physicians, nurses, and allied health professionals and ii) persons with PD and apathy and/or their family caregivers. This was done to understand how different stakeholder groups use knowledge, and to help ensure persons with PD and their family caregivers would feel comfortable sharing their opinions. All participants spoke English, were not aphasic, and were able to provide informed consent.

HCP were recruited via convenience sampling. Practitioners were included if they were a neurologist, psychiatrist, psychologists, nurse, or allied health professional within the Calgary Movement Disorder Clinic (MDC), and had experience managing apathy in at least one individual with PD. HCP were contacted via email.

Patients were recruited via a convenience sample from within the MDC. Patients were included if they had a diagnosis of idiopathic PD made by a movement disorder specialist and a Starkstein Apathy Scale (SAS) score of 14 or greater. The SAS has a sensitivity of 66% and specificity of 100% [22, 23]. Individuals were excluded if they had significant symptoms of depression based on a score of 9 or above on the short form of the Geriatric Depression Scale [24, 25] or evidence of significant cognitive impairment based on a score of 4 or less on the Memory Impairment Screen [26]. These assessments were administered via telephone, prior to recruitment. Family caregivers of included individuals were also invited to participate.

### Data collection and handling

Interview guides were developed with the aim of understanding what stakeholders perceive to be the barriers and facilitators to an optimal approach to diagnosing and managing apathy in PD. Interview guides were informed by the TDF, and a preceding scoping review of apathy diagnosis and management in PD. All guides were reviewed by members of the research team and granted approval for use by the CHREB.

One facilitator conducted focus groups (B.M.), with a note-taker (J.H-L. or Z.G.). One researcher facilitated interviews (B.M.). The researcher who facilitated focus groups and interviews (B.M.) recorded their own answers to the interview guide, to bracket researcher bias prior to beginning facilitation [27]. Focus groups and interviews were recorded using an audio recorder and transcribed verbatim, using notes taken during focus groups to assess reflexivity [27]. Upon completion of focus groups, debriefing discussions took place between the facilitator and note-taker and were documented. All transcripts had identifying factors removed and 10 % of transcripts were checked for consistency with audio recordings. Data were analyzed using NVivo (11.4.0).

### Analysis

Framework analysis was utilized to analyze transcripts. This analytic technique requires data to be summarized and classified into a thematic framework [21]. The thematic framework was based on the TDF and BCW, producing findings focused on practice-oriented results [21]. The TDF identifies 14 theoretical domains associated with the psychology of behavior change [28]. The BCW is concerned with how to successfully implement interventions by matching change interventions to behavioural barriers; an individual’s capability, opportunity, and motivation interrelate and influence their behaviour (COM-B) [29].

The primary researcher (B.M.) developed initial codes and themes for all transcripts. The first 25% of coding was reviewed iteratively with two members of the research team (Z.G. and H.H.). After this point, all coding was verified by Z.G. Three researchers (B.M., Z.G., and H.H.) assigned codes and themes to the TDF and BCW categories. These themes were then reviewed again by all four researchers (H.H., Z.G., J.H-L., and B.M.) to confirm all final coding and theme assignment.

## Results

### Participants (Tables 1 and 2 to be placed below this paragraph)

A total of two focus groups and eight interviews took place, including a total of 16 participants (11 females). If participants were unable to attend focus groups, interviews took place (Tables 1 and 2). HCP had experience ranging from < 1 year to 20 years. The percentage of persons with PD and apathy that the HCP had seen during their practice ranged from 5 to 70%. All persons with PD were recruited from the Calgary MDC. Ages ranged from 59 to 72 years, with PD duration ranging from 3 to 15 years. Duration of apathy ranged from 2 to 10 years.

**Table 1:** Description of Persons with PD and Caregivers Included

Focus Group or Interview	Person with PD, or caregiver, or HCP	Gender	Age	Duration of Parkinson’s Disease (years)	Duration of apathy (years)
Focus Group #1	Person with PD	M	61–65	1–5	2
Focus Group #1	Person with PD	F	56–60	11–15	10
Focus Group #1	Caregiver	M	61–65	NA	NA
Interview	Person with PD	F	71–75	6–10	2
Interview	Person with PD	F	66–70	1–5	4

**Table 2:** Description of HCPs Included

Interview	F	2	10
Interview	F	9	70
Interview	M	< 1	5
Interview	M	18	50
Interview	M	8	60
Focus Group #2	F	20	NR
Focus Group #2	F	32	10
Focus Group #2	F	20	15
Focus Group #2	F	15	20–30
Focus Group #2	F	NR	NR
Interview	F	NR	50

### Barriers and facilitators to diagnosis (Table 3 to be placed below this paragraph)

Words in parentheses below represent the TDF domain(s) associated with the given code.

**Table 3:** Barriers and Facilitators to the diagnosis of apathy in PD using the COM-B and TDF framework

COM-B System		TDF	Diagnosis Related Codes	Facilitator or Barrier
Capability	Psychological	Knowledge	A variety of screening tools are used to identify neuropsychiatric symptoms other than apathy	Facilitator
			Apathy as dynamic changing symptom	Barrier
			Apathy exists as a symptom isolated from other neuropsychiatric symptoms	Facilitator
			Apathy is often diagnosed with other neuropsychiatric symptoms	Facilitator
			Apathy presents in a variety of ways	Barrier
			Education on apathy as a symptom in PD aids health care practitioners in making a diagnosis	Facilitator
			Recognizable symptoms in those with apathy in PD	Facilitator
			Knowing about apathy facilitates diagnosis	Facilitator
			Lack of awareness of apathy as a symptom hinders diagnostic and management processes	Facilitator
			Lack of screening tools for apathy	Barrier
			Health care practitioner may be aware of apathy without making a formal diagnosis	Barrier
			Limited utility of screening tools if there is not one definition for apathy	Barrier
			Need to create increased awareness that apathy is a part of the non-motor symptoms in PD	Barrier
			Physicians require diagnostic process prior to providing management	Barrier
		Skills	A variety of screening tools are used to identify neuropsychiatric symptoms other than apathy	Facilitator
			Education on apathy as a symptom in PD aids health care practitioners in making a diagnosis	Facilitator
			Getting patient history helps health care practitioner make diagnosis	Facilitator
			Health care practitioners use varied diagnostic processes to identify apathy in PD	Barrier
			Recognizable symptoms in those with apathy in PD	Facilitator
			Knowing about apathy facilitates diagnosis	Facilitator
			Health care practitioner may be aware of apathy without making a formal diagnosis	Barrier
		Memory, attention and decision processes	A variety of screening tools are used to identify neuropsychiatric symptoms other than apathy	Facilitator
			Apathy exists as a symptom isolated from other neuropsychiatric symptoms	Facilitator
			Apathy is often diagnosed with other neuropsychiatric symptoms	Facilitator
			Family and persons with PD communicating symptoms and behaviour changes to physician aids diagnostic process	Facilitator
			Getting patient history helps health care practitioner make diagnosis	Facilitator
			Health care practitioners use varied diagnostic processes to identify apathy in PD	Barrier
			Recognizable symptoms in those with apathy in PD	Facilitator
		Behavioural Regulation	Family and persons with PD communicating symptoms and behaviour changes to physician aids diagnostic process	Facilitator
Opportunity	Physical	Environmental Context and Resources	Screening tools to overcome lack of knowledge or experience	Facilitator
			Lack of screening tools for apathy	Barrier
Motivation	Reflective	Beliefs about capabilities	A range of health care practitioners should be able to identify apathy to aid diagnosis	Facilitators
			Physicians require diagnostic process prior to providing management	Barrier
			Screening tools may interfere with physician expertise and judgment	Barrier
			Screening tools to overcome lack of knowledge or experience	Facilitator
			Inherent lack of engagement in those with apathy makes diagnosis difficult	Barrier
			Lack of awareness of apathy as a symptom hinders diagnostic and management processes	Barrier
			Limited utility of screening tools if there is not one definition for apathy	Barrier
		Social/professional role and identity	A range of health care practitioners should be able to identify apathy to aid diagnosis	Facilitator
			Lack of awareness of apathy as a symptom hinders diagnostic and management processes	Barrier
		Intentions	Apathy in care facility may purposefully go underreported	Barrier
		Beliefs about consequences	Apathy in care facility may purposefully go underreported	Barrier
			Need for a diagnostic tool for apathy	Barrier
		Optimism	Apathy symptoms are difficult to see	Barrier
		Goals	Need for a diagnostic tool for apathy	Barrier
			Need to create increased awareness that apathy is a part of the non-motor symptoms in PD	Barrier
	Automatic	Social/Professional Role and Identity	Screening tools may interfere with physician expertise and judgment	Barrier
			Family and persons with PD communicating symptoms and behaviour changes to physician aids diagnostic process	Facilitator
		Reinforcement	Family and persons with PD communicating symptoms and behaviour changes to physician aids diagnostic process	Facilitator
		Emotion	Inherent lack of engagement in those with apathy makes diagnosis difficult	Barrier

Apart from initial screening for inclusion within this study, patients included within this study stated they had never received a formal diagnosis of apathy (Knowledge, Skills) (Table 3). HCP reported they may be aware of apathy without making a formal diagnosis (Knowledge, Skills). It was identified that apathy is difficult to see and may present in a variety of ways (Optimism, Knowledge). While some HCP reported a screening tool may be helpful when ruling out comorbidities that present with apathy (Knowledge, Skills, Memory, attention and decision processes), overall it was reported there is limited use to screening tools for apathy if there is no formally accepted definition of apathy (Knowledge, Beliefs about capabilities).

“I personally was never diagnosed with apathy, I just know I have it.” PTC P1

“I’ve never been diagnosed with apathy either … There’s no formal diagnosis ever been offered of apathy” PTC P3

“There’s no definition or diagnostic criteria for apathy, that makes it virtually impossible to categorize. The scoping systems [reviews] can say well, you know, you scored kind of in the *I got apathy zone* … but there’s no real way to diagnose it” PTC P3

A key barrier to diagnosis identified was the lack of awareness of neuropsychiatric symptoms in PD, including apathy (Knowledge, Beliefs about capabilities, Social/professional role and identity). HCP identified the overall lack of engagement and insight among those with PD and apathy is an additional barrier to diagnosis (Beliefs about capabilities, Emotion). It was also reported that apathy may go purposefully unreported in care facilities because passive behaviour is favorable (Intentions, Beliefs about consequences).

“So, I think the lack of awareness is a big problem, not only for apathy, for all the non-motor symptoms of the disease.” HCP P2

“ … this state I’m in, I don’t think I was mentally prepared for this so like you say that might be a good, like had I known then it’s just like ok this is a possibility this may happen or along that line … ” Patient/Caregiver (PTC) P1

“ … people with apathy often come, with more severe apathy obviously, they come through as being … quite disengaged in the way they give an account of their day and … the type of emotional accompaniment to their speech is a red flag … they are not very collaborative … ” HCP P4

“ … they live in a care facility, and I think care facilities probably don’t mind apathy as much as other things and so the impetus then to report is quite a bit lower.” HCP P5

All included participants agreed there needs to be increased awareness of apathy as a symptom in PD (Knowledge, Goals). Increased education on apathy as a symptom in PD was thought to aid HCP in making a diagnosis (Knowledge, Skills). With some participants suggesting research applying focus groups and qualitative methods may aid in increasing awareness of apathy (Beliefs about consequences, Social influences). It was also reported that a variety of HCP should be able to identify apathy to facilitate diagnosis (Beliefs about capabilities, Social/professional role and identity).

“ … even if you can’t do anything about it, to create an understanding of it for patients and families … I’ve under appreciated the value of that, I think … and I probably still do. I think for families to say … we know what this is, it has a name, is really empowering for them.” HCP P5

“I think education about identifying, specifically targeting … being more aware of behaviours that might indicate apathy, having it more on the radar, maybe. I think depression tends to be up there but apathy it seems to be a little bit of a harder [symptom].” HCP P6

“ … it’s one [the diagnosis of apathy] that really should cut across a number of different specialties that work within the clinic and … I think the typical practitioner that makes it [the diagnosis of apathy] is probably the psychiatrist, or neuropsychiatrist that works in a clinic.” HCP P5

Facilitators to diagnosis included communication between HCP and family/persons with PD (Memory, attention and decision processes, Behavioural regulation, Social/professional role and identity, Reinforcement, Skills). Other facilitators to diagnosis were, knowing that apathy can exist as a symptom isolated from other neuropsychiatric symptoms or as a symptom along with other neuropsychiatric symptoms (Knowledge, Memory, attention and decision processes, Intentions, Beliefs about consequences).

“I don’t use it [screening tools] in the practice, I usually go based mostly on the family history … and I talk to the patient about this also because usually when they tell me about it, they can recognize some aspects [of apathy].” HCP P2

“Those who have apathy also have quite significant depression or cognitive impairment.” HCP P1

“I try and dispel misconceptions about it, particularly one of the more common misconceptions is that their relative is in fact depressed, and suffering from a sadness or a dislike of whatever the activities are.” HCP P5

“I started to notice that for depression I … feel that … you feel down, you feel teary. But apathy is totally different, apathy just is, you just don’t really want to get involved and really there’s nothing in my life that’s horrible so I couldn’t really understand why I felt so … you know disengaged, definitely disengaged.” PTC P5

### Barriers and facilitators to overall management (Table 4 to be placed below this paragraph)

Physicians reported they manage apathy based on experience versus evidence, as there are currently no guidelines for care of apathy in PD (Skills, Environmental context and resources) (Table 4). Furthermore, it was reported there is a lack of resources available pertaining to apathy treatment in PD (Knowledge, Environmental context and resources). A lack of evidence concerning apathy was part of a larger issue identified; overall there is limited information on neuropsychiatric symptoms of PD (Knowledge). This limits the capacity for HCP to provide treatment options to persons with PD (Beliefs about capabilities, Environmental context and resources).

“Again, there are not many guidelines, I really like evidence based medicine and there are not good guidelines for this [apathy]. The evidence is very poor.” HCP P2

“ … there’s a remarkable amount of literature that sort of describes like the 10 cardinal symptoms of PD and you know, this is what PD is and this is what PD patients do. There’s a significant … lack of attention to this whole complex of motivation, apathy, joy, reward … if they [newly diagnosed person with PD] haven’t been told that this [apathy] could be a significant problem, they’re certainly lead astray … ” PTC P3

[On the biggest barrier to managing apathy in PD] “ … probably not enough facilities, or not enough capacity for all allied health professionals to take on this problem” HCP P4

“We need a pathway of care that is focused on apathy, we need an algorithm of treatment that makes the approach more standardized and helps interaction with other health professionals.” HCP P4

Pharmacologic options for treatment are often considered a first line intervention or a quick fix, with most information coming from control trials (Knowledge, Memory, attention, and decision processes, Environmental context and resources). Often, it was reported that the treatment of other neuropsychiatric symptoms that present with apathy aids management (Knowledge, Skill, Memory, attention and decision processes).

“ … one quick way to try and improve it [apathy] would be pharmacological. So, and I gauge their acceptance of adding on a medication of that type.” HCP P4

“I am not aware of any treatments that specifically target apathy outside of the context of where it occurs with other diagnoses. So, I have seen apathy improve in patients with cognitive impairment who go on … cholinesterase inhibitors. And in patients with depression I have seen apathy improve with antidepressants but also with stimulant medications.” HCP P1

**Table 4:**
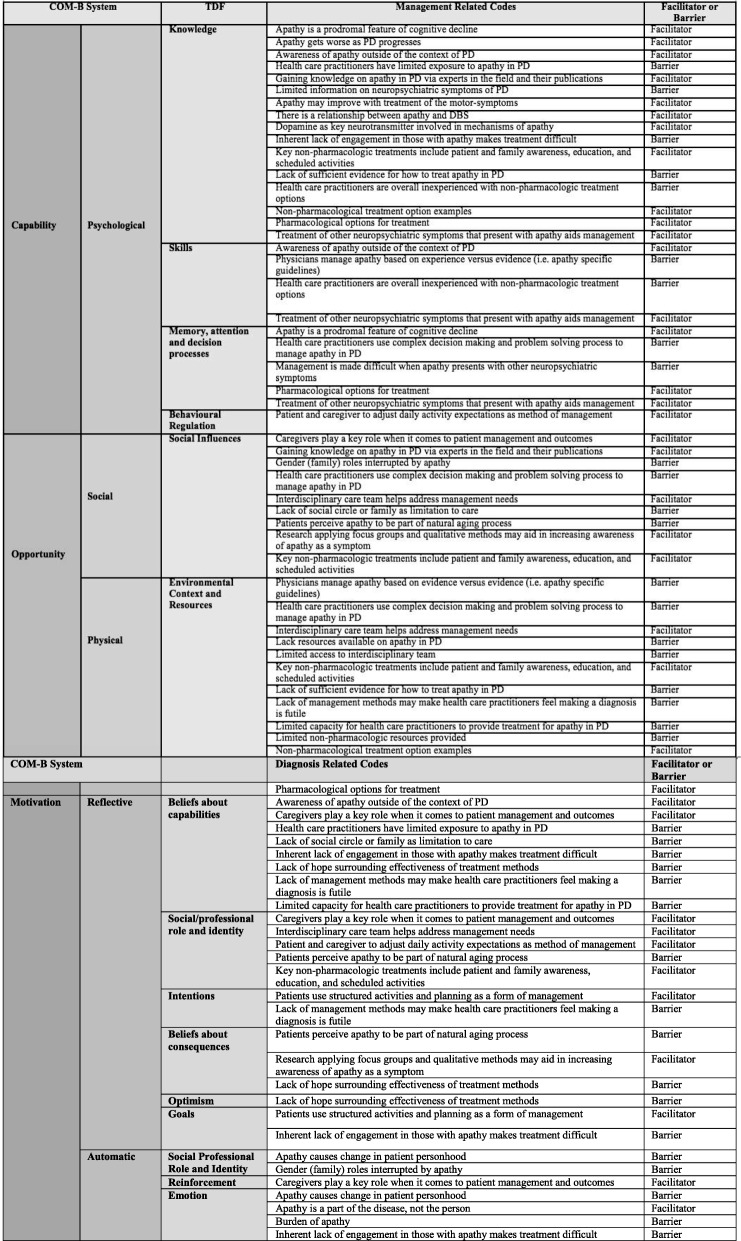
Barriers and Facilitators to the management of apathy in PD using the COM-B and TDF framework.

However, persons with PD prefer non-pharmacologic interventions and feel there are limited non-pharmacologic resources provided (Environmental context and resources). HCP reported that overall, they are inexperienced with non-pharmacologic treatment (Knowledge, Skill).

“ … whereas personally the less medication you’re on the better I think but if there are tools you know for example … you mention exercise in your … non-prescription medication [s] [to] treat apathy aren’t extensive but would be worthwhile I think.” PTC P3

HCP reported the lack of evidence for management contributed to feeling a sense of futility in making the diagnosis (Beliefs about capabilities, Environmental context and resources, Intentions). HCP also described a lack of hope surrounding the effectiveness of treatment methods for apathy in PD (Beliefs about capabilities, Beliefs about consequences, Optimism). However, persons with PD and their caregivers preferred to receive a diagnosis of apathy, with awareness and education on apathy reported as key techniques for management (Knowledge, Social/professional role and identity, Environmental context and resources, Social influences).

“I also think the treatment refractory nature of it [apathy] … pharmacologically, I think a lot of physicians have the mindset, if you can’t fix it with a drug then I don’t know why they’re seeing me for it.” HCP P5

“I don’t have a strong level of hope that any treatment is really going to make a big difference for people.” HCP P1

“I find that some of the neurologists are a little bit hesitant to discuss kind of mental components of PD, apathy being one of them, motivation etc.” PTC P3

All participants reported family caregivers play a key role when it comes to patient management and outcomes (Social/professional role and identity, Beliefs about capabilities, Social influences, Reinforcement). It was also identified that interdisciplinary care teams can address management needs (Social/Professional Role and Identity, Environmental Context and Resources, Social Influences). However, our findings suggest there is limited access to interdisciplinary teams (Environmental context and resources).

“They almost need like a surrogate outside of their brain to do that thing, that, that part of the brain is no longer doing to really initiate the activity. And going back to that idea of inertia, that once you get something moving it’s a lot easier to move. And so, encouraging families to be persistent in inviting their loved one out to do things … ” HCP P5

“But since I’ve mentioned it to my [child], [they have] been trying to, as I say get me out more.” PTC P4

“So, my ideal maximum care would have a very nice team … with people working particularity in mood aspects that are not depression. That is why I was saying maybe a psychologist, or maybe a spiritual councillor. Someone that can talk about different things...” HCP P2

“ … you see I’ve been 15 years, I’ve been diagnosed [with PD] and I just saw a psychiatrist [for the] first time last month.” PTC P1

Patients and their caregivers reported apathy causes a change in personhood (Social/professional role and identity, Emotion). However, an important aspect to living with apathy in PD is recognizing that apathy is a part of the disease and not the person (Emotion). Some persons with PD believed apathy was a natural part of aging (Social/professional role and identity, Social influences, Beliefs about consequences). Persons with PD and apathy also described the large burden of living with apathy (Emotion). HCP noted the importance of persons with PD and family caregivers adjusting daily activities and expectations as a method of management (Social/professional role and identity, Behavioural regulation).

“ … yeah, things that came naturally before they just don’t come naturally now, now its planning like you have to plan. like you really have to think things out ABC whereas before you just sort of did stuff” PTC P1

“I keep giving books to my children and going here read this pamphlet, you need to understand what she’s going through so you have a better understanding why she’s looking at you like this and … She’s not being mean or … It’s just not her” PTC P2

“I, while I found it odd I thought well you know maybe this is just part of the aging process … ” PTC P3

“ … whereas the motor symptoms of the disease are frustrating, I find personally this inextricably linked motivation, reward, joy, apathy complex that exist … the most frustrating part of the disease” PTC P3

“ … we start to talk about other resource that the caregiver may need so that they can care for themselves … we may start talking with them about the importance, about taking better care of themselves and … I don’t mean letting go but recognizing they need to care for themselves...” HCP P9

## Discussion

In order to understand how to develop an optimal approach to apathy in PD we examined what key stakeholders perceived to be barriers and facilitators to the diagnosis and management of apathy in PD. Framework analysis based on the TDF allows our findings to inform the development of guidelines and interventions [28].

A major theme surrounding diagnostic barriers was the lack of a standardized apathy definition, which limits any possibility for improvement of diagnostic processes. This was clearly expressed by all participants. While validated diagnostic criteria exist and have been suggested for use within the clinical setting, these criteria were not utilized by HCPs in our study population, nor had any persons with PD and apathy been aware or diagnosed with apathy in the clinic [7, 8].

Our preceding scoping review came to a similar conclusion; the current lack of a standardized definition of apathy limits our ability to evaluate the effectiveness of available apathy screening tools against gold standard criteria [19, 30]. While available screening tools have demonstrated diagnostic validity, future research should aim to improve overall quality of evidence by first establishing clear definitions of neuropsychiatric symptoms, such as apathy [30].

Interestingly, a recent study assessing barriers and facilitators to the use of clinical practice guidelines for depression and anxiety in PD or dementia identified similar themes [31]. Overlapping themes included a lack of evidence on depression, anxiety and apathy in PD, lack of awareness and availability of screening tools, and concerns regarding symptomatic overlap [31]. These findings suggest that even where neuropsychiatric symptoms are more understood and where guidelines for HCP exist, the quality and breadth of literature on non-motor symptoms in general, is limited. Future studies should acknowledge and address gaps established by such qualitative research, as these gaps limit the quality of current and future clinical practice guidelines.

A recent study employed the TDF to assess barriers to help seeking for non-motor symptoms in persons with PD [32]. Barriers included patient’s lack of knowledge that non-motor symptoms were related to PD, a belief that there were limited treatment options for non-motor symptoms, and a hesitancy to add any pharmacologic interventions to their management plan [32]. These themes closely align with our findings that show patients with PD did not perceive apathy to be a part of PD but rather the natural aging process. Additionally, our findings identified patients and family caregivers have limited treatment options and prefer non-pharmacologic treatments to pharmacologic treatment. Taken together, these studies demonstrate a need for increased awareness that PD is more than a movement disorder. Furthermore, research on non-pharmacological interventions for non-motor symptoms should be given priority, due to it being a preferred method of treatment for end-users.

Interestingly, stakeholders identified the use of qualitative research to better understand apathy in PD as an important area for future research. Currently, there is only one other study that employs qualitative methods, with ours being the second within the literature [20]. This research identified methods for managing apathy in PD should revolve around behavioural interventions and the support of social circles [20]. Both qualitative studies identified that, while HCP may be hesitant to make patients and family caregivers aware of apathy without possibility of treatment, communication regarding the meaning and behavioural implications of this symptom are necessary [20].

Future research may also utilize qualitative methods to better understand how persons with PD and their caregivers conceptualize apathy. This may help inform a universally accepted definition of apathy. Furthermore, future qualitative research studies should be conducted specifically discussing key barriers identified within this research, most importantly the lack treatment methods. Given our findings, that persons with PD and their caregivers prefer non-pharmacological treatment, qualitative research may inform the development of more targeted and preferred treatment options.

Limitations of our study include the small sample size of patients and caregivers. This may in part be explained by our strict inclusion criteria, to ensure only those with pure apathy were included. While the sample size was small, saturation occurred such that no new codes were generated following our final focus groups/interviews, in each stakeholder group. This may also in part be explained by the specificity of the topic of investigation [21]. Additionally, we combined interview data with focus group data, to ensure all eligible participants were included regardless of scheduling conflicts. We noted data collected in both interviews and focus groups was similar. Specifically, similar experiences were described concerning overall lack of an official diagnosis of apathy given to persons with PD, the importance of awareness, and the lack of available treatment options, reported by all participants. Our patient sample was relatively young, with a mean age of 66 and primarily included females. Therefore, our population may not be representative of older persons with PD or the primarily male population effected by PD [33]. Furthermore; we excluded individuals with depression and mild cognitive impairment. Often, these symptoms occur with apathy, and thus may limit generalizability of our research. Additionally, as participants were required to contact our study if they were interested in participating, we may have missed recruiting the most apathetic persons with PD due to the inherent nature of this symptom.

## Conclusions

These findings suggest that the process of diagnosing apathy may be improved with increased awareness of the symptoms. Managing apathy in PD requires interdisciplinary teams, which also include family caregivers. We identified that where HCPs perceive lack of knowledge as a barrier to making a diagnosis, persons with PD and family caregivers find just being given a diagnosis facilitates overall understanding. Barriers reported suggest future research must aim to identify a standard definition for apathy, along with validated screening tools and specific treatments, with priority given to non-pharmacologic treatment options.

## Abbreviations

BCW: Behaviour Change Wheel
CHREB: Conjoint Health Research Ethics Board
COM-B: Capability, Opportunity, Motivation, Behaviour
HCP: Health Care Practitioner
MDC: Movement Disorder Clinic
PD: Parkinson’s disease
PTC: Patient and caregiver
SAS: Starkstein Apathy Scale
TDF: Theoretical Domains Framework
